# Oxidative Stress Protection by Canary Seed (*Phalaris canariensis* L.) Peptides in Caco-2 Cells and *Caenorhabditis elegans*

**DOI:** 10.3390/nu14122415

**Published:** 2022-06-10

**Authors:** Uriel Urbizo-Reyes, Kee-Hong Kim, Lavanya Reddivari, Joseph M. Anderson, Andrea M. Liceaga

**Affiliations:** 1 Protein Chemistry and Bioactive Peptides Laboratory, Purdue University, 745 Agriculture Mall Drive, West Lafayette, IN 47907, USA; uurbizor@purdue.edu; 2 Department of Food Science, Purdue University, 745 Agriculture Mall Drive, West Lafayette, IN 47907, USA; keehong@purdue.edu (K.-H.K.); lreddiva@purdue.edu (L.R.); 3 Department of Agronomy, Purdue University, 915 W. State St., West Lafayette, IN 47907, USA; janderson@purdue.edu

**Keywords:** canary seed peptides, *Caenorhabditis elegans*, oxidative stress, antioxidant potential

## Abstract

During oxidative stress, degenerative diseases such as atherosclerosis, Alzheimer’s, and certain cancers are likely to develop. Recent research on canary seed (*Phalaris canariensis*) peptides has demonstrated the high in vitro antioxidant potential. Thus, this study aimed to assess the cellular and in vivo antioxidant capacity of a low-molecular-weight (<3 kDa) canary seed peptide fraction (CSPF) using Caco-2 cells and the *Caenorhabditis elegans* model. The results show that the CSPF had no cytotoxicity effect on Caco-2 cells at any tested concentration (0.3–2.5 mg/mL). Additionally, the cellular antioxidant activity (CAA) of the CSPF was concentration-dependent, and the highest activity achieved was 80% by the CSPF at 2.5 mg/mL. Similarly, incubation with the CSPF significantly mitigated the acute and chronic oxidative damage, extending the lifespan of the nematodes by 88 and 61%, respectively. Furthermore, it was demonstrated that the CSPF reduced the accumulation of reactive oxygen species (ROS) to safe levels after sub-lethal doses of pro-oxidant paraquat. Quantitative real-time PCR revealed that the CSPF increased the expression of oxidative-stress-response-related gene GST-4. Overall, these results show that the CSPFs relied on GST-4 upregulation and scavenging of free radicals to confer oxidative stress protection and suggest that a CSPF can be used as a natural antioxidant in foods for health applications.

## 1. Introduction

Hairless canary seed (*Phalaris canariensis* L.) is a novel cereal grain that has gained the attention of scientists due to its high protein content (23%), unique starch chemistry, and potential application as a functional ingredient [1,2,3,4]. Typical canary seeds are not safe for human consumption due to the presence of toxic siliceous hairs on the surface of the seeds’ kernel; however, crossbreeding techniques allowed the development of novel hairless varieties that are safe and approved for human consumption [5]. Previous studies demonstrated the high in vitro antioxidant activity of canary seed extracts, and it was believed that phenolic compounds were responsible for their antioxidant activity. However, recent research efforts have linked the antioxidant activity of canary seeds to the release of hydrophobic peptides from prolamin proteins during gastrointestinal digestion [6,7]. In accordance with these findings, various studies have also shown that protein hydrolysis is associated with a rise in antioxidant properties [8,9,10,11]. Moreover, it has been shown that a peptides’ rise in antioxidant activity is dependent on their amino acid composition, structure, hydrophobicity, and position in the peptide sequence [12]. For instance, amino acids such as tyrosine, tryptophan, methionine, lysine, cysteine, and histidine are known for their high antioxidant activity attributed to their hydrogen-donating, peroxyl-radical-trapping, and/or metal-ion-chelating abilities [13,14]. Furthermore, regarding the amino acid residue position, tyrosine in the C- and N-termini are important motifs of antioxidant peptides of rice (*Oryza sativa*) bran protein [15]. Likewise, the presence of non-polar residues such as valine, leucine, isoleucine, alanine, and phenylalanine at the N-terminal was a common characteristic of antioxidant peptides from ostrich (*Struthio camelus*) egg whites [16]. In this context, it is imperative to study antioxidant peptides derived from novel protein sources with high nutritional and biological value to satisfy the rising population’s food demand and preference for naturally sourced ingredients.

Under normal physiological conditions, DNA and human cells undergo exposure to reactive oxygen species (ROS) derived from essential metabolic processes in the human body or compounds commonly present in our environment, such as X-rays, ozone, smoke, environmental pollutants, and industrial chemicals [17]. This natural process repeats regularly; nevertheless, this balance becomes unfavorable as we age. Its progression contributes to aging and the development of several chronic complications, such as neurodegenerative disorders, atherosclerosis, inflammation, and certain cancers [18]. Generating canary seed peptides with commercial proteases could be an excellent strategy for developing bioactive agents that could mitigate the damage caused by ROS in the body [6,7]. Yet, the understanding of the health benefits of canary seed peptides remains limited, and no evidence has shown that their antioxidant activities could be translated *in vivo*. Thus, using an appropriate in vivo model organism to study oxidative stress is critical to recapitulate any metabolic implication of canary seed peptide supplementation. 

In this respect, the nematode *Caenorhabditis elegans* is widely used as a cost-effective animal model to study pharmacological strategies to treat or prevent various human diseases and their effects at metabolic or genomic levels. Recently, *C. elegans* was used as a model to elucidate genetic pathways related to the antioxidative and anti-aging properties of phenolic compounds from mulberry (*Morus alba*) [19], and bioactive peptides from saltwater clam (*Meretrix meretrix*) [20]. In addition, it has been estimated that around 50% of *C. elegans* genes are homologous to genes implicated in human diseases, and studies have demonstrated that *C. elegans* is a valuable in vivo system for studying oxidative stress response pathways [21]. Therefore, this study aimed to evaluate the cellular (using Caco-2 cells) and in vivo (using a *C. elegans* model) antioxidant effect of canary seed peptides to generate new knowledge on the potential molecular mechanisms of these bioactive peptides involved in lowering oxidative stress.

## 2. Materials and Methods

### 2.1. Materials

USDA-GRAS hairless canary seeds (CDC Cibo) were purchased from a commercial vendor (Canpulse Foods LTD, Saskatoon, SK, Canada). Alcalase^®^ (protease from Bacillus licheniformis, P2.4 U/g) was purchased from Novozymes (Bagsvaerd, Denmark). Nematode N2 (wild-type) *C. elegans* and bacterial strain Escherichia coli OP50 were purchased from the Caenorhabditis Genetics Center (University of Minnesota, Minneapolis, MN, USA). Ascorbic acid, 3-(4,5-dimethylthiazolyl-2)-2,5-diphenyltetrazolium bromide (MTT), tert-butyl hydrogen peroxide (t-BOOH), methyl viologen dichloride hydrate (paraquat), Eagle’s Minimum Essential Medium (EMEM), and Caco-2 cells were all purchased from Millipore Sigma (St. Louis, MO, USA). Chemicals and materials not specified above were purchased from companies VWR International (Radnor, PA, USA), Millipore Sigma (St. Louis, MO, USA), and Thermo Fisher Scientific (Waltham, MA, USA).

### 2.2. Preparation of Canary Seed Peptide Fraction (CSPF) 

Canary seeds were prepared as previously reported [6]. Briefly, a canary seed protein solution (22.5 mg of protein/mL at pH 8) was hydrolyzed at 50 ± 3 °C for four hours with 3% (*w/w*) Alcalase^®^. Proteolysis was stopped by pasteurization (95 ± 3 °C) for 15 min. Then, the solution was cooled and centrifuged (17,636× *g* for 15 min) (Avanti J-26S Centrifuge, Beckman-Coulter INC. Brea, CA, USA). The supernatant was collected as a whole protein hydrolysate (WPH) and stored at −80 °C until use. The WPH was subject to simulated gastrointestinal digestion (SGD) using the method described by You, Zhao [22]. A WPH solution (10 mg of protein/mL, at pH 2) was prepared using 1 M HCl and then incubated with pepsin (4% weight/weight of protein) at 37 °C for two hours. The pH was increased to 5.3 using a 0.9 M NaHCO_3_ solution and increased further to 7.5 using a 1.0 M NaOH solution. Pancreatin was then added (4% *w/w* of protein), and the mixture was incubated again at 37 °C for two hours. SGD was terminated by pasteurization (95 ± 3 °C) for 15 min. Subsequently, the solution was centrifuged at 11,000× *g* for 15 min. The supernatant was ultrafiltered using a <3 kDa cutoff membrane to collect the peptide fraction identified in our previous study as being the most bioactive [6], which was referred to as canary seed peptide fraction (CSPF). CSPF was frozen at −80 °C for 12 h and freeze-dried using a Labconco FreeZone Plus 2.5 L cascade benchtop freeze dry system (Labconco Corp., Kansas City, MO, USA). Lastly, CSPF concentrations used in this study were based on previous investigations [6], preliminary antioxidant assays in Caco-2 cells, and *C. elegans* model.

### 2.3. Proximate Composition

CSPF was analyzed for moisture, ash, lipid, and protein content following the AOAC methods 950.46(b), 920.153, 960.39, and 984.13 (A-D), respectively (AOAC 2016), through a commercial analytical laboratory (A&L Great Lakes, Fort Wayne, IN, USA).

### 2.4. Total Amino Acid Analysis

The total amino acid analysis of CSPF digesta was performed by the University of Missouri Agriculture Experiment Station Chemical Laboratories (University of Missouri, Columbia, MO, USA) as previously described [23]. Briefly, two hundred milligrams of CSPF were subjected to complete hydrolysis with 6 N HCI at 155 °C for 16 h. The amino acids were analyzed using high-performance, cation exchange resin column in the Beckman 6300 Amino acid Analyzer (Beckman Instruments, Fullerton, CA, USA).

### 2.5. Cellular Viability Test

The cytotoxicity of CSPF was tested using the MTT assay as proposed by Er, Koparal [24]. Briefly, Caco-2 cells (passage 18) were seeded on a 96-well culture plate at 1 × 10^4^ cells/well and grown for 24 h using Dulbecco’s Modified Eagle Medium (DMEM). The DMEM was removed, and a 100 µL aliquot of DMEM containing 0.31, 0.62, 1.25, or 2.50 mg/mL of CSPF was added to the cells and incubated for 24 h at 37 °C in 5% CO_2_ conditions. Then, CSPF medium was removed, and 100 µL of an MTT solution (0.5 mg/mL) in EMEM was added to the cells. After two hours of incubation in dark conditions at 37 °C in 5% CO_2_, 100 µL of dimethylsulfoxide (DMSO) was added to dissolve blue crystals. Finally, the optical density was measured at 570 nm using Multiskan™ FC Microplate Photometer (Waltham, MA, USA). Cell viability was expressed as a percentage of viable cells relative to control cells (untreated cells).

### 2.6. Cellular Antioxidant Activity (CAA)

The CAA was evaluated following a modified methodology by Malaypally, Liceaga [25]. Briefly, 100 µL of Caco-2 cells (passage 18) were placed in a black 96-well plate (2.5 × 10^5^ cells/mL) and incubated for 6 h under 5% CO_2_ at 37 °C. Then, the growth medium was removed, and the cells were washed using 1× phosphate-buffered saline (PBS). Next, the cells were incubated with 100 µL of DMEM with 60 µM dichlorodihydrofluorescein diacetate (DCFH-DA) for one hour, followed by a washing step, and then exposed to a solution of CSPF (0.31, 0.62, 1.25, or 2.50 mg/mL) in DMEM for an additional hour. The CSPF solution was removed from each well, followed by a final wash with 1x PBS. Then, the cells were exposed to an oxidizing environment by adding 100 µL of 500 µM 2,2′-azobis (2-amidinopropane) dihydrochloride (AAPH) solution to each well. Dichlorodihydrofluorescein (DCFH) production was measured every 5 min using the fluorescent reader Spectra Max Gemini EM spectrofluorometer (Molecular Devices, Sunnyvale, CA, USA) with an excitation wavelength of 485 nm and an emission wavelength of 538 nm. The sample blank contained DMEM and DCFH-DA without AAPH; negative control wells contained cells with DCFH-DA and AAPH, and the positive control wells contained cells treated with L-ascorbic acid (50 µM), DCFH-DA, and AAPH. The cellular antioxidant activity was calculated by measuring the area under the curve based on Equation (1).

CAA unit = 100 − (ʃSample A/ʃblank A) × 100(1)

### 2.7. Caenorhabditis Elegans Growth and Maintenance

*C. elegans* was grown and maintained according to the methodology proposed by Bai, Farias-Pereira [26] and Solis and Petrascheck [27]. Briefly, 0.7 mL of *E. coli* (OP50) grown overnight in lysogeny broth (LB) medium was plated on nematode growth medium (NGM) agar plates. Immediately after, wild-type (N2) strains of *C. elegans* were seeded, and the plates were incubated at 25 °C. After 2–3 days, enough nematode concentrations were achieved, and the worms were collected by washing with M9 buffer. The OP50 was removed by three washing steps, in which the supernatant was discarded after 10 min of sedimentation. Consequently, the worms were synchronized by suspending them in 700 µL of M9 buffer with 300 µL of household bleach, and 200 µL of 5 M NaOH, followed by 5 min of vigorous vortexing. The eggs were then collected by centrifugation 3000× *g* for 2 min and washed three times with M9 buffer. After the third wash step, eggs were suspended in 10 mL and hatched in a sterile test tube. Furthermore, L1-stage worms were grown to early L4 stage by incubating them in an NGM plate with live *E. coli* (OP50) for 44–46 h. Finally, L4 nematodes were collected and utilized for further experimentation. 

### 2.8. Chronic and Acute Oxidative Stress Using C. elegans Model

The effect of CSPF on chronic and acute oxidative stress of *C. elegans* was determined following the methodology proposed by Bai, Farias-Pereira [26] and Zhao, Cheng [28]. Briefly, nematodes in the L4 stage were incubated in S-complete medium solution with CSPF (1, 2, or 3 mg/mL), 5-Fluoro-2′-deoxyuridine (FUdR) (120 μM), carbenicillin (2.8 μg/mL), and amphotericin (0.40 μg/mL) for 24 h at 25 °C. After incubation with CSPF, chronic or acute oxidative stress assessment was carried out. For chronic stress, a solution of S-complete-containing paraquat (25 mM), FUdR (120 μM), carbenicillin (2.8 μg/mL), amphotericin (0.40 μg/mL), and 20% of LB broth with *E. coli* (OP50) was added to the upper (200 μL), and lower (600 μL) compartment of a 24-transwell plate, and the media were changed every 70 h. The chronic stress analysis was initiated by adding 25 worms/well. For acute oxidative stress, 100 μL of a solution of S-complete-containing t-BOOH (5 mM) and 20% of LB broth with *E. coli* (OP50) was added to wells containing 25 worms/well using a 96-well plate. A total of 100 worms per treatment were scored microscopically based on their movement every 30 min for acute oxidative stress and 12 h for chronic oxidative stress, counting live and dead nematodes to determine their resistance to oxidative stress.

### 2.9. Quantitative Analysis of the Intracellular Reactive Oxygen Species (ROS)

The quantification of intracellular ROS accumulation in animals was carried out using the method by Sarasija and Norman [29]. Briefly, L4 nematodes were pre-treated with CSPF (1, 2, or 3 mg/mL) for 24 h as described in Section 2.8. Then, the worms were washed three times with M9 buffer and incubated in a solution of S-complete-containing paraquat (50 mM), FUdR (120 μM), carbenicillin (2.8 μg/mL), amphotericin (0.40 μg/mL), and 20% of LB broth with *E. coli* (OP50) for a period of 48 h at 25 °C. After that, L4 nematodes were washed three additional times with M9 buffer and then suspended in PBS at a pH 7 with 0.05% Tween^®^20. Next, worms were lysed by pulsed sonication for 40 s in an iced bath using a QSonica Q500 Sonicator (Qsonica LLC, Newton, MA, USA). An aliquot (50 μL) of worm extract (200 μg of protein/mL) was pipetted into a black 96-well plate with 50 μL of DCFH-DA (500 μM). The plate was incubated at 37 °C, and the ROS levels were quantified after 45 min using the fluorescent reader Spectra Max Gemini EM spectrofluorometer (Molecular Devices, Sunnyvale, CA, USA) at an excitation wavelength of 485 nm and an emission wavelength of 538 nm.

### 2.10. Gene Expression by Quantitative Real-Time Polymerase Chain Reaction (qPCR)

RNA was extracted following the methodology proposed by Green and Sambrook [30], and the qRT-PCR analysis followed the methodology proposed by Ogawa, Kodera [31]. Briefly, L4-stage nematodes were treated for 24 h with CSPF (3 mg/mL) at 20 °C as indicated in Section 2.5. Subsequently, the RNA was extracted by homogenizing nematodes in 1 mL of TRIzol (Invitrogen) for 5 min. RNA was then purified by adding 150 μL of chloroform followed by centrifugation at 12,000× *g* for 15 min at 4 °C. The supernatant was recovered as a source of RNA and was further washed and centrifuged three times with 0.5 mL of 75% ethanol at 12,000× *g* for 3 min. Finally, the pellet was resuspended with 0.5 mL of DPEC water, 0.5 mL of isopropanol, and 50 µL of a 3 M sodium acetate solution at pH 5.5 followed by centrifugation at 12,000× *g* for 15 min at 4 °C. The precipitated RNA was then suspended in DEPC water, and complementary DNA (cDNA) was subsequently synthesized using the superscript II kit according to the manufacturer’s protocol. cDNA was amplificated using SYBR premix Plus (SYBR Green) (Bio-Rad Laboratories, Hercules, CA, USA). The reaction was carried out using StepOne Real-Time PCR System (Applied Biosystems, Foster City, CA, USA), and the expression was normalized to housekeeping gene β-actin. Lastly, the data were analyzed using the Delta Delta CT (2^−∆∆Ct^) method to calculate the fold gene expression change relative to the control (no peptide exposure). Primers used to quantify each transcript are specified in Supplementary Table S1.

### 2.11. Statistical Analysis

The results in this study were analyzed using a complete randomized design by a one way-ANOVA followed by a Tukey’s post hoc test (*p* < 0.05 and *p* < 0.01). In addition, a log-rank methodology was applied for survival estimates at (*p* < 0.01 and *p* < 0.0001). The statistical analysis was carried out using the statistical software JMP^®^ PRO version 15.1.0 (SAS Institute, Inc., Cary, NC, USA). At least three independent experiments were performed for each assay, and in the case of *C. elegans* studies, a minimum of three different populations, with at least 100 nematodes per treatment, were utilized. Finally, the results are reported as mean ± standard deviation (SD) of triplicate determinations.

## 3. Results and Discussion

### 3.1. Proximal Composition and Amino Acid Analysis

The CSPF was composed of carbohydrate 74% (by difference), protein 22%, ash 3%, and fat 1% (*w/w*, dry basis) content. Compared to previously reported raw canary seed powder consisting of carbohydrate 71% (by difference), protein 18%, ash 6%, and fat 5% (*w/w*, dry basis) [6], the CSPF was higher in carbohydrate and protein but lower in ash and fat. The amino acid composition of the CSPF is shown in Table 1. In particular, the CSPF had a higher content of glutamic acid 30.7%, isoleucine 4.3%, and histidine 2.0% compared to raw unhydrolyzed canary seed protein containing 26.0, 3.9, and 1.6%, respectively [5]. Concerning other cereal proteins, glutamic acid in the CSPF was also higher than that of rice (19%), maize (18%), barley (23%), oat (21%), and rye (24%) [1,32]. Furthermore, the concentration of aromatic amino acids (phenylalanine, tyrosine, and tryptophan) in the CSPF (13%) was also superior to that of maize (9%), rice (10%), wheat (8%), barley (10%), oat (10%), and rye (8%) [32]. In addition, the CSPF had a higher concentration of arginine (6%) and isoleucine (4%) than those present in maize, wheat, barley, and oat [32]. Although the CSPF was deficient in methionine and lysine, the level of essential amino acids was 34%, comparable to that reported for other cereal sources, which ranged between 29 and 35%, and remained constant when compared to unhydrolyzed canary seed protein [32]. While no specific antioxidant peptide sequences were identified in the present study, we previously identified and reported peptides in this CSPF that were rich in polypeptides (4–8 amino acids long) and contained a high content of glutamine, proline, and cationic residues (histidine and arginine) [33]. In this respect, proteolysis with commercial proteases such as Alcalase could contribute to the exposure of radical scavenging residues (e.g., aromatic, cationic, and non-polar amino acids) that increase the antioxidant activity and promote the rise in the glutamic acid content observed in this study. Nevertheless, future research should focus on developing a detailed identification of amino acid sequences in the CSPF involved in these antioxidant properties.

### 3.2. Effect of Canary Seed Peptides on Cellular Viability and Oxidative Stress

**Figure 1:**
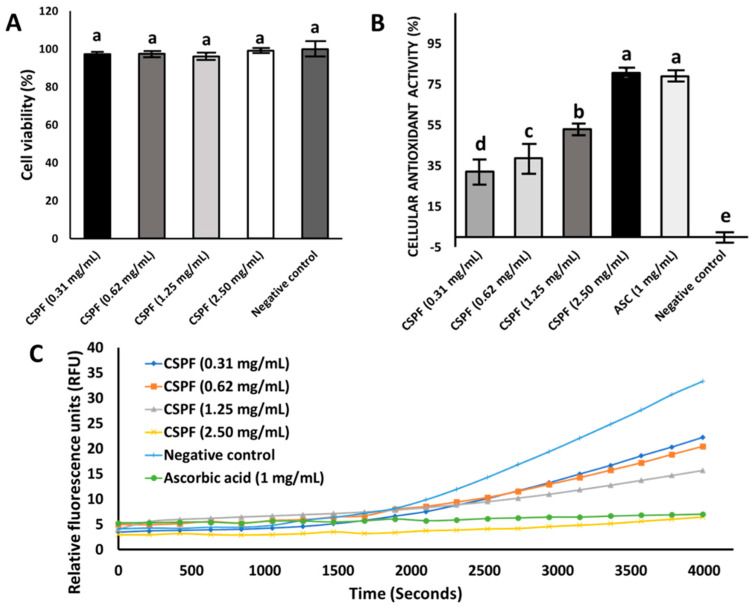
(**A**) Cell viability (%): MTT assay over canary seed peptide fraction (CSPF) at different protein concentrations, (**B**) intracellular antioxidant activity (%) of CSPF and ascorbic acid (ASC), and (**C**) inhibition of peroxyl-radical-induced DCFH oxidation to DCF by CSPF and ASC. Negative control: untreated cells. Bars and lines represent mean values of triplicate determinations ± standard deviation. Different letters (a–e) indicate statistical differences (*p* < 0.05) between samples.

Caco-2 cells were selected in this study for their ability to provide a more robust association with active membrane transport (bioavailability) in the intestinal cell wall compared to other cell types (e.g., L-929, HepG2) [34,35]. Cellular viability assessment by the MTT assay is a well-recognized methodology and used on a wide range of molecules (e.g., drugs, topical ingredients, proteins, and phytochemicals) to assess the toxicity of an agent [36]. In this study, we evaluated the viability of Caco-2 cells after 24 h of exposure to the peptide fraction (CSPF) at various concentrations (Figure 1A). Results show that the CSPF was not cytotoxic at any of the tested concentrations (1–3 mg/mL), where no significant difference (*p* > 0.05) was found among tested concentration levels, and the cell viability ranged between 96 and 100%. Additionally, cell confluency remained unaffected, and cell loss was not observed in this study. Even though no information was available regarding CSPF cellular toxicity, these results align with observations reported in other toxicological studies, where no adverse effects were seen when feeding rats with a 50% hairless canary seed diet [37]. Subsequently, we analyzed the cellular antioxidant activity of the CSPF (Figure 1B). We monitored the oxidation of the intracellular probe DCFH-DA to its fluorescent counterpart DCF after exposure to the CSPF for one hour (Figure 1C). The probe DFH-DA is introduced to the cell and subject to deacetylation by cellular esterases; this leads to the production of the oxidable form of DCFH to DCF. Thus, for an antioxidant to prevent oxidation of DCFH to DCF, it must permeate the cell and compete intracellularly with free radicals from AAPH. Our results show that the CSPF confers a homogenous and gradual cellular antioxidant protection that increases significantly with concentration (*p* < 0.05), offering cellular antioxidant activity (CAA) of up to 80% for the CSPF at 2.5 mg/mL. There were no significant differences between the CSPF (2.5 mg/mL) and the positive control ascorbic acid (1 mg/mL). The high CAA observed in this study could be linked with the low molecular weight (<3 kDa) and the high content of aromatic amino acids 13% (*w*/*w* of protein) present in the CSPF (Table 1). Aromatic amino acids such as tyrosine, phenylalanine, and tryptophan are well-known for their excellent proton donation capabilities, stabilizing electron-deficient radicals, and preventing oxidative damage [14,38]. From a cellular transport perspective, we previously reported that CSPF had a high cellular transport capacity (>10%); this property could also contribute to their ability to scavenge radicals at intracellular levels [33].

### 3.3. Antioxidant Properties of Canary Seed Peptides against Chronic and Acute Oxidative Stress

**Figure 2:**
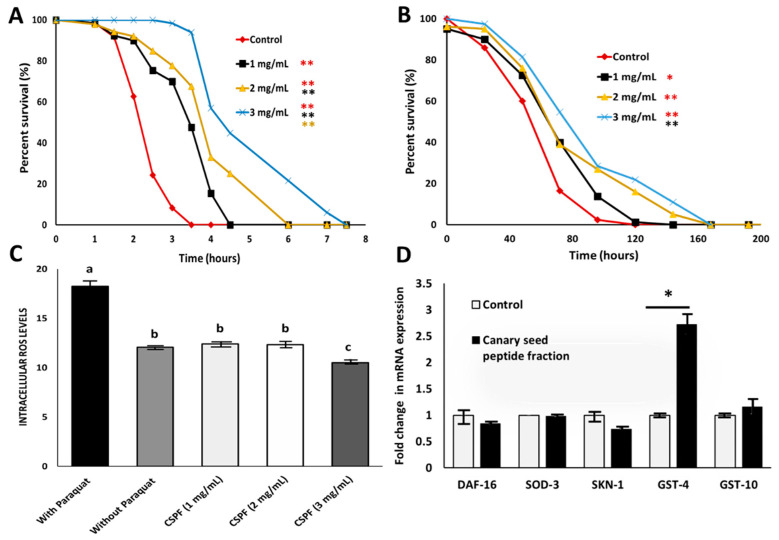
In vivo antioxidant activity of canary seed peptide fraction (CSPF), using a *C. elegans* model. (**A**) Survival analysis of *C. elegans* pre-exposed to CSPF for 24 h followed by acute oxidative stress induction with tert-butyl hydrogen peroxide (t-BOOH). (**B**) Survival analysis of *C. elegans* pre-exposed to CSPF for 24 h followed by chronic oxidative stress induction with paraquat. (**C**) Intracellular accumulation of reactive oxygen species (ROS) in *C. elegans* after pre-exposure to CSPF for 24 h followed by paraquat for 48 h. (**D**) Relative mRNA levels of expression of DAF-16, SOD-3, SKN-1, GST-4, and GST-10 after 24 h exposure with 3 mg/mL of CSPF using ACT-1 as an internal control. Bars and lines represent mean values ± standard deviation. Three independent experiments were performed with at least 100 nematodes per treatment. Statistical differences are indicated as * *p* < 0.01, and ** *p* < 0.001 or different letters (a–c) *p* < 0.01.

This study explored if CSPF have molecular implications for oxidative stress using a *C. elegans* model. Based on the cellular toxicity analysis, CSPF (1, 2, and 3 mg/mL) were used to assess their in vivo antioxidant activity. When pre-exposed to the CSPF, followed by induction of acute oxidative stress using t-BOOH, CSPF treatments were shown to significantly (*p* < 0.001) increase *C. elegans*’s lifespan compared to nematodes exposed to S-complete buffer alone (Figure 2A). The antioxidant protection was shown to be concentration-dependent, and the lifespan was extended significantly (*p* < 0.001) by 45, 63, and 88% at 1, 2, and 3 mg/mL peptide concentrations, respectively. These results are similar to those observed for the cellular antioxidant activity (Figure 1B), indicating an equally high capacity to permeate and protect the cells from acute oxidative damage in whole organisms. In the case of chronic oxidative stress (Figure 2B), CSPF pre-exposure was successful in significantly (*p* < 0.05) extending the lifespan of *C. elegans* relatively to the control by 23, 42, and 61% for the 1, 2, and 3 mg/mL peptide concentrations, respectively. The impact on the survival rate (%) derived from CSPF pre-exposure was lower chronic oxidative stress compared to acute oxidative stress; this could be due to the half-life of the antioxidant peptides, whose effects tend to diminish over time due to protein turnover [39]. Notably, on the chronic stress survival analysis, a significant difference between CSPF samples was only detected between 1 and 3 mg/mL. In this regard, these results are in agreement with those observed for ROS quantification (Figure 2C), where the treatment with 3 mg/mL decreased ROS levels (*p* < 0.01) compared to the 1 and 2 mg/mL treatments. Similarly, Zhang, Jiang [40] showed that supplementation of green alga (*Chlorella vulgaris*) protein hydrolysates at a concentration of 4 mg/mL successfully lowered the ROS in *C. elegans* compared to the untreated nematodes or lower peptide concentrations (1–2 mg/mL). We previously demonstrated the in vitro antioxidant capacity of peptides derived from commercial hydrolysis of canary seed protein [6]; yet, to the best of our knowledge, this is the first time the antioxidant activity of canary seed peptides obtained from simulated gastrointestinal digestion has been tested *in vivo*, indicating their nutraceutical potential. From these results, we confirm that the antioxidant capacity of a CSPF relies on the scavenging of free radicals as one of the mechanisms by which it prevents acute and chronic oxidative stress at both cellular (Caco-2) and live-organism (*C. elegans*) levels.

### 3.4. Expression of Antioxidant-Related Genes 

The expression of various antioxidant-related genes (DAF-16, SOD-3, SKN-1, GST-4, and GST-10) was monitored by quantitative real-time PCR (qPCR) after 24 h of exposure to a high dose (3 mg/mL) of a CSPF in order to determine which molecular mechanisms could be tied to the antioxidant potential of a CSPF applied to *C. elegans*. The results for gene expression fold change are shown in Figure 2D. After pre-treatment with the CSPF, there was no significant difference in gene expression for DAF-16, SOD-3, SKN-1, or GST10. Nevertheless, significant upregulation was found for the GST-4 gene. GST-4 encodes the antioxidant enzyme glutathione S-transferase, which is involved in suppressing ROS formation and confers longer survival time to nematodes [41]. Research has demonstrated that glutathione-S-transferases (GSTs) act in the second detoxification phase, reducing ROS into less toxic compounds [42]. It has been reported that some plant metabolites have induced the expression of GST-4 and other enzymes involved in glutathione biosynthesis and metabolism. For instance, Pohl, Teixeira-Castro [43] and Ma, Cui [44] showed that GST-4 was upregulated after incubation of *C. elegans* with rapeseed extract and sesame peptides, respectively. In another study, tyrosol, a simple phenol from olive oil, was an effective inducer of GST-4 and promoted a drop in ROS levels of treated *C. elegans* nematodes [45]. Finally, other plant-derived compounds have shown that flavonoids such as baicalein, chrysin, and 6-hydroxyflavone were effective in extending the *C. elegans* longevity via GST-4 induction [46]. Interestingly, the GST-4 upregulation by CSPF was shown to be independent of the SKN-1 signaling pathway, an upstream regulator of antioxidant and detoxification genes such as GST-4. It is possible that GST-4 induction could be happening via the epidermal growth factor (EGF) signaling pathway and is promoted by EOR-1; this could also explain the lack of transcriptional activity from other GSTs such as GST-10 [47]. In this context, others have reported that royalactin, a honeybee protein, increased the expression of GST-4 via the EPG signaling pathway with no impact on the SKN-1 gene [48]. It is hypothesized that CSPFs could increase EGF expression, resulting in EOR-1 translocation to the nucleus, and promote the transcription of the detoxification gene GST-4, which might help decrease oxidation and extend *C. elegans*’s survival to oxidative stress [49]. Nevertheless, further investigations applying EGF- or SKN-lacking mutants as well as targeted analysis of protein expression levels are necessary to confirm this hypothesis. Overall, the results of this study suggest that GST-4-mediated antioxidant activity in coordination with the scavenging of free radicals could be the reason for the extended nematode survival times observed in this study.

## 4. Conclusions

In this study, we demonstrated that a canary seed peptide fraction produced by commercial enzymatic proteolysis with Alcalase followed by simulated gastrointestinal digestion showed no cytotoxic effect on Caco-2 cells at any of the tested concentrations (1–3 mg/mL). In addition, pre-exposure to a CSPF significantly lowered oxidative damage in Caco-2 cells in a concentration-dependent matter. Furthermore, we showed that pre-exposure to the canary seed peptide fraction substantially increased the resistance to acute and chronic oxidative stress by extending the lifespan of a live organism (*C. elegans*). The results from this study reveal that the CSPF relies on radical scavenging and up-regulation of the anti-oxidative-related gene (GST-4) to confer antioxidant protection to *C. elegans*, demonstrating the feasibility of using this simple in vivo model to establish preliminary determination of the antioxidant activity of peptides. Finally, GST-4 modulation was shown to be independent of upstream signaling pathways such as SKN-1 and suggests an alternative antioxidant route may be involved. Further research will be needed to elucidate this possibility and the biological activity that peptide fractions from canary seed might offer to more complex model organisms. Taken all together, the results in this study demonstrate that canary seed peptides can confer antioxidant protection in vivo and can be valuable as a nutraceutical ingredient for the food and pharmaceutical industries. Future steps should be focused on developing a more detailed understanding of specific peptide sequences responsible for the antioxidant activity observed in this study.

## Figures and Tables

**Table 1:** Total amino acid composition of canary seed peptide obtained from simulated gastrointestinal digestion.

Amino Acids	Relative Content (g/100 g)
Taurine §	0.10
Aspartic Acid	4.79
Threonine	2.50
Serine	3.98
Glutamic Acid	30.72
Proline	6.34
Lanthionine §	0.19
Glycine	3.21
Alanine	4.44
Cysteine	2.50
Valine	4.55
Methionine	1.37
Isoleucine	4.31
Leucine	7.56
Tyrosine	3.46
Phenylalanine	6.34
Hydroxylysine	0.30
Ornithine §	0.07
Lysine	2.00
Histidine	2.02
Arginine	6.27
Tryptophan	2.98
AAA	12.78
PCAA	10.29
SAA	3.88
HAA	41.08
EAA/NEAA	0.51

Results are expressed on a dry basis. §: non-proteinogenic amino acids. AAA: aromatic amino acids. PCAA: positively charged amino acids. SAA: sulfur-containing amino acids. HAA: hydrophobic amino acids. EAA/NEAA: essential to non-essential amino acid ratio.

## Data Availability

Not applicable.

## Supplementary Materials

The following supporting information can be downloaded at: https://www.mdpi.com/article/10.3390/nu14122415/s1. Table S1: Primer sequences used.
