# Supplementary material for: Oxidative Stress Protection by Canary Seed (Phalaris canariensis L.) Peptides in Caco-2 Cells and Caenorhabditis elegans
Source: Nutrients. 2022 Jun 10;14(12):2415. doi: 10.3390/nu14122415 (PMC9227596; doi:10.3390/nu14122415)
Supplement: Supplementary file 1 [file nutrients-14-02415-s001.zip › nutrients-1747966-supplementary.pdf]

Table S1. Primer sequences used.

Primer	Sequence
act-1 forward	TCCAAGAGAGGTATCCTTAC
act-1 reverse	CGGTTAGCCTTTGGATTGAG
daf-16 forward	CTTCAAGCCAATGCCACTACC
daf-16 reverse	GGAGATGAGTTGGATGTTGATAGC
skn-1 forward	GACGTCAATTTATGGAGTGTCG
skn-1 reverse	GAAGATGTTTTGTCGTGATCCG
sod-3 forward	CCAACCAGCGCTGAAATTCAATGG
sod-3 reverse	GGAACCGAAGTCGCGCTTAATAGT
gst-4 forward	TGG AGA CTC ATT GAC TTG GG
gst-4 reverse	TCC TTT CTT GTT GCC ACG
gst-10 forward	CGT GCC ACA ACT TTA CTA CTT C
gst-10 reverse	CAA CTG ACC AAG GAG CAT TC
